# The complete chloroplast genome of *Alhagi sparsifolia* Shap. (Leguminosae)

**DOI:** 10.1080/23802359.2021.1944366

**Published:** 2021-06-30

**Authors:** Xi Jiang, Wen-Juan Huang, Ya-Rui Li, Jie Si, Jing-Dong Xu, Er-Dai Qin, Tian-Ge Yang, Hong Liu, Zhi-Hua Wu, Pei-Pei Jiao

**Affiliations:** a College of Plant Science, Tarim University, Alar, China; b Xinjiang Production and Construction Corps Key Laboratory of Protection and Utilization of Biological Resources in Tarim Basin, College of Life Science, Tarim University, Alar, China; c Hubei Provincial Key Laboratory for Protection and Application of Special Plant Germplasm in Wuling Area of China, College of Life Sciences, South-Central University for Nationalities, Wuhan, China; d Foreign Exchange and Cooperation Department, Tarim University, Alar, China; e College of Life Science and Technology, Huazhong Agricultural University, Wuhan, China

**Keywords:** *Alhagi sparsifolia*, Leguminosae, chloroplast, genome, evolution

## Abstract

*Alhagi sparsifolia* Shap. is a perennial herbaceous plant belonging to the genus *Alhagi*, Leguminosae. This species is of high nutritional, medicinal and ecological values. The complete chloroplast genome was 128,418 bp and lost an IR (inverted repeat) region. Further annotation revealed the chloroplast genome contains 108 genes, including 75 protein coding genes, 29 tRNA genes, and 4 rRNA genes. A total of 103 simple sequence repeats (SSRs) were identified in the chloroplast genome. This chloroplast genome resource will be useful for study on the evolution and genetic diversity of *A. sparsifolia* in the future.

*Alhagi sparsifolia* Shap., is a perennial herbaceous plant belonging to the genus *Alhagi*, Leguminosae. It is widely distributed in arid desert areas of China, such as Inner Mongolia, Gansu, Qinghai and Xinjiang province. It provides plentiful crude protein, crude fiber, and unsaturated fatty acids for livestock as an ideal forage. A series of pharmacologically active secondary metabolites, such as flavonoids, alkaloids, steroids, pseudalhagin A, phospholipids, and polysaccharides have been extracted from *Alhagi* species (Muhammad et al. 2015), and they show broadly biological activities in antioxidant, cardiovascular, anti-ulcer, hepatoprotective, antispasmodic, antidiarrheal, antinociceptive, antipyretic, anti-inflammatory, anti-rheumatic, antibacterial, and antifungal (Muhammad et al. 2015; Zhou et al. 2017). Besides, *A. sparsifolia* also plays an important role in stabilizing and improving the fragile ecological environment in desert areas as a typical xerophyte. Therefore, *A*. *sparsifolia* has a series of nutritional, medicinal and ecological values.

In this study, to obtain a new insight into the phylogeny of *A. sparsifolia*, we sequenced, assembled, and annotated the accurate chloroplast genome of *A. sparsifolia*. The materials of *A. sparsifolia* in this study was collected from Wushi City, Aksu Prefecture, Xinjiang province of China (79°1′0.12″E, 41°7′1.8″N, 1580 m above the sea level). The complete genomic DNA was extracted using the modified CTAB method (Doyle and Doyle 1987) and sequenced using the Illumina NovaSeq platform at Majorbio Company (Shanghai, China). The raw reads were generated and low-quality sequences were filtered out. The trimmed reads were assembled using GetOrganelle (Jin et al. 2020). The assembled genome was annotated using CPGAVAS2 (Shi et al. 2019) and PGA (Qu et al. 2019). The complete chloroplast genome was 128,418 bp (MW349013) and lost an IR region, the average GC content was 34.05%. Previous studies have examined the phylogenetic distribution of different plastid genome rearrangements among legumes, and found the loss of one copy of the IR from the tribes Carmichaelieae, Cicereae, Hedysareae, Trifolieae, Fabeae, Galegeae, and three genera of Millettieae (Palmer and Thompson 1982; Lavin et al. 1990; Liston 1995; Jansen et al. 2008). The chloroplast genomes encoded 108 functional genes, including 75 protein coding genes, 29 tRNA genes, and 4 rRNA genes. A total of 103 SSR markers ranging from mononucleotide to hexa-nucleotide repeat motif were identified in *A. sparsifolia* chloroplast genome.

To explore the phylogenetic relationship of *A. sparsifolia* within Leguminosae, the complete chloroplast genomes of 23 species from Leguminosae were obtained from the GenBank database, with the *Polygala fallax* Hemsl. and *Polygala tenuifolia* Willd. as the outgroups, and the phylogenetic trees were built from the whole protein-coding gene matrix by maximum-likelihood (ML) and Bayesian inference (BI) (Figure 1). The ML tree was generated using IQ-TREE (Nguyen et al. 2015) based on the best model of TVM + F + I + G4 and 1000 bootstrap replicates, and BI analysis was performed in MrBayes-3.2.7 (Ronquist et al. 2012). The results show the analyzed *A. sparsifolia* was similar to other Galegeae species, which all lack an IR region. The *A. sparsifolia* was closer to the species of *Tibetia* and *Caragana*. This information will be useful for study on the evolution and genetic diversity of *A. sparsifolia* in the future.

**Figure 1.**
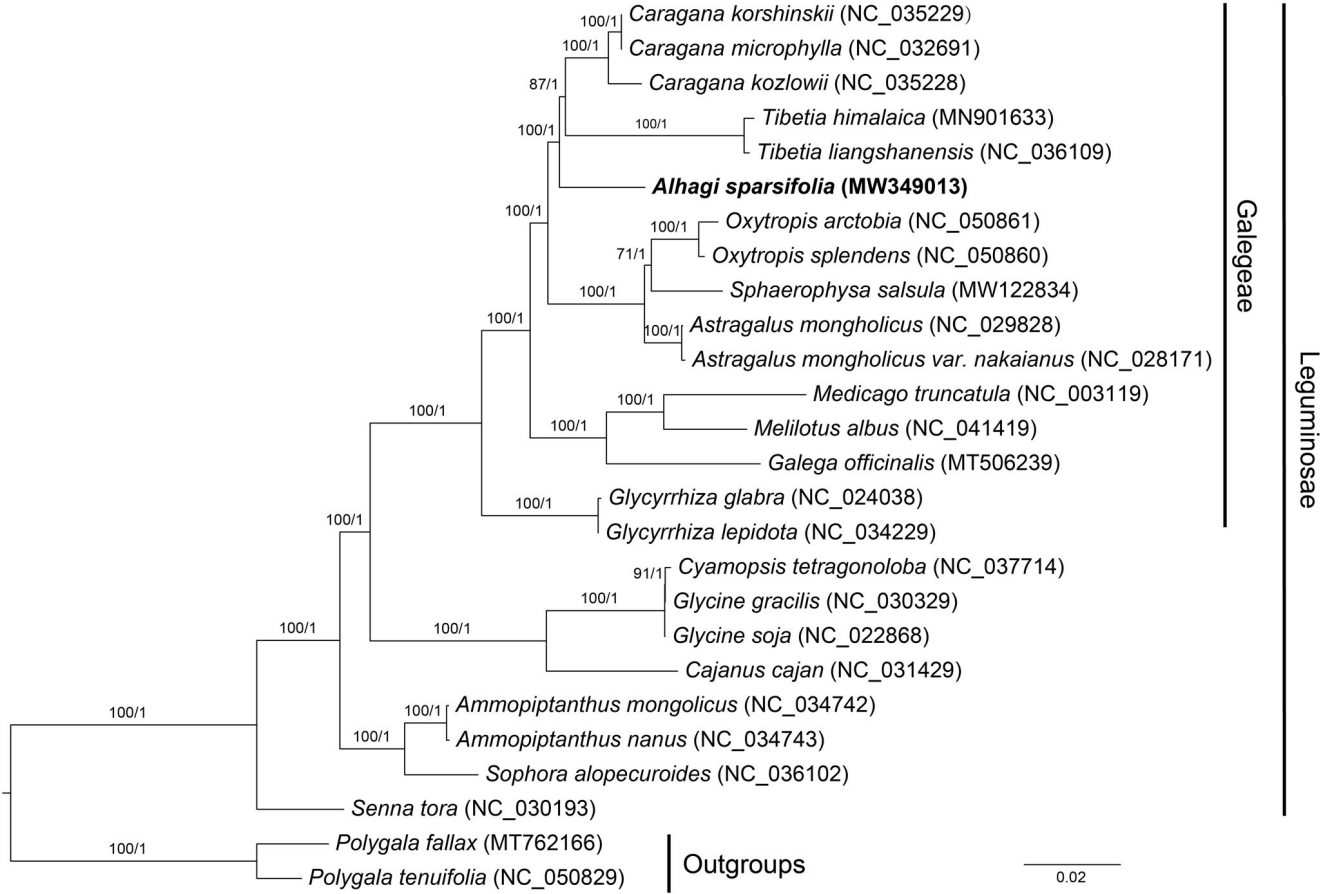
Phylogenetic tree reconstructed by maximum-likelihood (ML) and Bayesian inference (BI) analysis based on the whole chloroplast protein-coding genes of these 26 species. The numbers on each branch represent the bootstrap value for ML method and posterior probability for BI method, respectively.

## Data Availability

The specimen and DNA sample were deposited at the herbarium of Tarim University (https://www.taru.edu.cn/, Wen-Juan Huang, hwjzky@163.com) under the voucher number TD-00558. The genome sequence data that support the findings of this study are openly available in GenBank of NCBI at [https://www.ncbi.nlm.nih.gov] (https://www.ncbi.nlm.nih.gov/) under the accession no. MW349013. The associated “BioProject”, “SRA”, and “Bio-Sample” numbers are PRJNA685338, SRR13255665, and SAMN17083245 respectively.
